# Public awareness of obesity causes, complications, and treatment methods in a representative sample of adults in Poland

**DOI:** 10.3389/fpubh.2025.1656877

**Published:** 2025-09-18

**Authors:** Kuba Sękowski, Agnieszka Mazurek, Zuzanna Grześczyk-Nojszewska, Mateusz Jankowski, Agnieszka Kamińska, Agata Olearczyk, Andrzej Silczuk, Justyna Grudziąż-Sękowska

**Affiliations:** ^1^School of Public Health, Centre of Postgraduate Medical Education, Warsaw, Poland; ^2^Ophthalmology Department, Faculty of Medicine, Collegium Medicum, Cardinal Stefan Wyszyński University in Warsaw, Warsaw, Poland; ^3^Health Innovation Unit, SGH Warsaw School of Economics, Warsaw, Poland; ^4^Department of Environmental Psychiatry, Faculty of Life Sciences, Medical University of Warsaw, Warsaw, Poland

**Keywords:** obesity, health literacy, disease awareness, socioeconomic factors, health knowledge, attitudes, diet, lifestyle

## Abstract

**Introduction:**

Obesity is a growing public health problem. This study aimed to assess public awareness of obesity causes, complications, and treatment methods in a representative sample of adults in Poland.

**Methods:**

A cross-sectional study was conducted in May 2025 among a representative sample of 1,088 Polish adults using a computer-assisted web interview (CAWI). The structured questionnaire assessed attitudes toward obesity with 10 different questions.

**Results:**

Most respondents (84.8%) correctly identified obesity as a disease. Lifestyle factors—lack of physical activity (82.4%) and unhealthy diet (73.9%)—were the most frequently reported causes. Complications such as type 2 diabetes (81.1%) and hypertension (79.2%) were well recognized, but awareness of conditions like polycystic ovary syndrome (17.3%) and asthma (24.7%) was limited. Increased physical activity (86.9%) and diet (86.7%) were widely acknowledged as treatment methods, while fewer participants recognized pharmacotherapy (34.9%) or bariatric surgery (51.6%). Gender, education, and self-reported economic status significantly (*p* < 0.05) influenced awareness patterns. In multivariable analysis, female gender was associated with higher recognition of obesity as a disease (aOR: 1.62; 95%CI: 1.15–2.27; *p* = 0.005), while adults aged 30–39 showed lower recognition (aOR: 0.59; 95%CI: 0.37–0.96; *p* = 0.04).

**Conclusions:**

There is an urgent need for tailored public health education in Poland that emphasizes the multifactorial nature of obesity, addresses knowledge gaps in treatment options, and reduces social stigma. Strategic interventions must consider demographic and socioeconomic differences to improve population-level obesity literacy and outcomes.

## 1 Introduction

Obesity is a chronic complex disease characterized by excessive adiposity, which can impair health (1). In the majority of cases, it is considered to be multifactorial (2); however, the etiology is still at least partially unknown (3). Among its causes are obesogenic environments, high-calorie intake, psychological factors, inadequate physical activity, genetic variants, and diseases that affect the endocrine system. As a surrogate marker, BMI is used as a measure of adiposity (1).

From a public health perspective, obesity is now a pandemic and a global public health problem. The prevalence of obesity worldwide has nearly tripled since 1975 (4). In 2022, 12.5% of the world's population, or 890 million people, were obese (5). The prevalence of obesity is highest in the United States and lowest in Sub-Saharan Africa and Southeast Asia (4).

Obesity is also linked to socioeconomic status: in low-income countries, obesity is associated with higher socioeconomic status, while in highly developed countries; it typically correlates with lower socioeconomic status (6). This association is stronger for women than for men (6).

Until recently, obesity therapy included mostly attempts to reduce body weight through diet and physical activity and bariatric surgery. In recent years, the introduction of new drugs to the pharmaceutical market and the expansion of indications for existing drugs to other diseases have significantly increased the role of pharmacotherapy in treating obesity (7, 8). For many patients who did not have the opportunity or were afraid of surgical treatment, it opened up access to therapies that can radically improve their quality of life and functioning in society. In public opinion, this sudden increase in the popularity of treatment is sometimes even referred to as a “fashion” for treating obesity, often perceived very negatively (9).

Efforts are being made worldwide to reduce the incidence of obesity through educational activities, preventive measures, health programs, and appropriate legislative changes. Educational and preventive activities most often include educational classes in schools, cooking workshops, sports classes, and dietary consultations (10, 11). Legislative changes are mainly aimed at reducing demand and limiting the availability of high-calorie, high-sugar, high-fat, and highly processed foods. This is achieved by imposing additional taxes or fees on these products and increasing their prices, which is intended to translate into reduced demand while discouraging corporations from producing them (12).

Obesity prevention is also a key goal of health policies at the international level, including those of the European Union and the WHO, as well as in their plans and recommendations. The EU4 Health Program (13) provides member states with funding for health initiatives and the improvement of healthcare systems, with the overarching goal of reducing cancer incidence. One of the key elements of the program is Europe's Beating Cancer Plan (14), which promotes the reduction of cancer risk factors, with a particular emphasis on obesity and low physical activity. Improving diet and increasing physical activity are among the actions recommended in the program to be implemented from 2021 to 2025.

In 2022, the WHO adopted the Acceleration Plan to Stop Obesity (12), which sets ambitious goals for member states in obesity prevention by indicating specific tasks to be achieved by 2025 and 2030. These goals are essential for the WHO's planned 30% reduction in premature deaths caused by non-communicable diseases by 2030 (15). The objectives include reducing the incidence of obesity among children, adolescents, and adults; decreasing the level of physical inactivity; promoting breastfeeding; increasing the availability of nutrition professionals; and introducing national regulations on advertising food and beverages to children.

Since it has been demonstrated that inequalities in income and educational levels among family members have a significant impact on the risk of overweight and obesity in young children (16, 17), this perspective must also be reflected in the planning of health policies. Such actions are possible, among others, by providing access to free, healthy food in schools and kindergartens, sports activities for seniors or promoting breastfeeding (18).

In the context of Poland, based on data from the Central Statistical Office from 2019 (19) for the population over 15 years of age, approximately 19.5% of men and 17.6% of women have obesity, and 45.6% of men and 31.3% of women are overweight. Over the last 15 years, there has been a consistent increase in the incidence of excess body weight. Obesity in Poland is most common among people over 50 years of age. Obesity and overweight as health problems in Poland began to intensify during the transformation of the political system as a result of dynamic socioeconomic changes and the resulting rapid adaptation to the Western lifestyle. Currently, the most disturbing trend in Poland is the rate of growth of obesity and overweight, especially in the youngest age groups (20).

Evaluating the public knowledge about obesity is an essential element in planning and implementing public policies and public health interventions. Observing changing paradigms about obesity (7, 21) allows, above all, more effective interventions to reduce the incidence and increase the availability and willingness to undertake treatment. Classifying and understanding obesity as a disease entity is necessary to reduce stigmatization and discrimination (2, 22) of people living with obesity and to improve their physical and mental health.

This study aimed to public awareness of obesity causes, complications, and treatment methods in a representative sample of adults in Poland.

## 2 Material and methods

### 2.1 Study design and population

This cross-sectional study was conducted in Poland between May 23 and May 26, 2025. A representative sample of the adult population was surveyed using a computer-assisted web interview (CAWI) questionnaire. The survey was administered by the Nationwide Research Panel Ariadna (23), a private research panel provider.

Participants were recruited from a panel of over 100,000 registered individuals. Quota sampling was employed to ensure stratification of the sample by age, sex, and place of residence, based on the demographic characteristics of the general population reported by Statistics Poland (24). Panelists who refused to participate were replaced with individuals from the corresponding stratum to ensure the minimum sample size of 1,000 respondents was met. Only complete responses were collected.

Participation in the survey was voluntary, and all responses were anonymous. Informed consent was obtained from each participant before administering the survey. The study was conducted in accordance with the principles outlined in the Declaration of Helsinki. The study protocol was approved by the Ethics Committee at the Center of Postgraduate Medical Education on May 14, 2025 (decision no. 41/2025).

### 2.2 Measures

The study questionnaire was prepared based on the literature review (3–10). All participants were required to complete a ten-item questionnaire and provide information regarding their height, weight, marital status, household size, number of children, education level, occupational status, and economic status. Height and weight data were used to compute Body Mass Index (BMI) and group respondents into four BMI categories (underweight: < 18.5, healthy weight: ≥18.5 to < 25, overweight: ≥25 to < 30, obesity ≥30) (25). The questionnaire was designed by the authors following a literature review (2–7). Four questions assessed the respondents' knowledge of obesity as a medical condition, and six questions examined their social perception of obesity. Only the questions regarding knowledge were explored in this publication. The remaining questions will be addressed in a subsequent publication.

One question assessed the respondents' recognition of obesity as a disease, with responses recorded on a five-point Likert scale ranging from “definitely yes” to “definitely no.” Responses of “definitely yes” and “rather yes” were aggregated to represent a “yes” response. Three check-all-that-apply questions, each with seven choices, assessed the respondents' knowledge about the causes, complications, and treatment and management of obesity.

A pilot study with 15 adults (general population, non-medical workers) was conducted to verify the questions used in the questionnaire. The study questionnaire was filled out 7 days apart. After this procedure, responses provided twice by the same group of respondents were analyzed, including content validation or stylistic check. One question was removed, two questions were modified, and four multiple-choice answers were revised.

### 2.3 Statistical analysis

Data analysis was conducted using IBM SPSS v.29 (Armonk, NY, United States). Categorical variables were presented in tables using raw counts and proportions. The associations between the demographic and socioeconomic characteristics of the respondents and their responses were evaluated using the chi-square test of independence. Logistic regression was used to explore the relationship between 10 demographic and socioeconomic independent variables and the recognition of obesity as a disease. Only the variables demonstrating a statistically significant relationship with the outcome in univariable regression were included in the multivariable regression model. The strength of the relationship was quantified using odds ratios (OR) and 95% confidence intervals (95% CI). The *p-value* of less than 0.05 defines statistical significance.

## 3 Results

One thousand eighty-eight adults participated in the study. Of those, 54.0% were female. In terms of age distribution, 30.1% were aged 60 years or older, 19.8% were aged 30 to 39, and 19.4% were aged 40 to 49. Over half of the study population was overweight (37.9%) or obese (20.2%). A total of 39.2% of participants were classified as having a healthy weight. A comprehensive summary of the study population's characteristics is presented in Table 1.

**Table 1:** Characteristics of the study population (*n* = 1,088).

**Variable**	***n***	**%**
**Gender**		
Female	588	54.0
Male	500	46.0
**Age group [years]**		
18–29	145	13.3
30–39	215	19.8
40–49	211	19.4
50–59	190	17.5
60+	327	30.1
**BMI category**		
Underweight	30	2.8
Healthy weight	426	39.2
Overweight	412	37.9
Obesity	220	20.2
**Education level**		
Higher	506	46.5
Less than higher	582	53.5
**Marital status**		
Single	289	26.6
Married	577	53.0
Informal relationship	180	16.5
Other	42	3.9
**Place of residence**		
Rural area	415	38.1
City below 20,000 residents	144	13.2
City from 20,000 to 99,999 residents	212	19.5
City from 100,000 to 499,999 residents	182	16.7
City ≥ 500,000 residents	135	12.4
**Having children**		
Yes	702	64.5
No	386	35.5
**Number of household members**		
1 (living alone)	171	15.7
2	418	38.4
3 or more	499	45.9
**Occupational status**		
Active	668	61.4
Passive	420	38.6
**Self-reported household economic status**		
Good	540	49.6
Moderate	399	36.7
Bad	149	13.7

### 3.1 Public knowledge of obesity

The majority of the study participants (“definitely yes”: 42.6% and “rather yes”: 42.2%) correctly identified obesity as a disease (Table 2). The most frequently selected causes of obesity were a lack of physical activity (82.4%), an unbalanced diet (73.9%), hormonal disorders (69.0%), and a genetic predisposition (66.2%) (Table 2). Type 2 diabetes was the most commonly recognized complication of obesity (81.1%). Additionally, 79.2% of respondents were aware that hypertension could result from obesity, and 73.5% understood that heart failure might also be a consequence. Notably, only 24.7% of respondents indicated asthma as a potential complication of obesity, and just 17.3% recognized polycystic ovary syndrome (PCOS) as a possible complication (Table 2). The two most identified methods for managing obesity were increased physical activity (86.9%) and a healthy diet (86.7%). Less than half of the respondents recognized that obesity management could involve psychological or psycho-dietetic interventions (43.1%), and even fewer (36.1%) were aware that regular sleep could be a management strategy. Furthermore, 51.6% of respondents knew that obesity could be treated surgically, while only 34.9% recognized pharmacotherapy as a treatment option (Table 2).

**Table 2:** Public knowledge of obesity in a representative sample of adults in Poland (*n* = 1,088).

**Variable**	***n***	**%**
**Do you think obesity is a disease?**		
Definitely yes	464	42.6
Rather yes	459	42.2
Rather no	71	6.5
Definitely no	16	1.5
I do not know	78	7.2
**What do you think may be the causes of obesity?**		
Poorly balanced diet	804	73.9
Lack of physical activity	897	82.4
Mental disorders, experiencing difficult emotions	572	52.6
Unfamiliarity with the principles of healthy eating	609	56.0
Lack of time for preparing nutritious meals and physical activity	527	48.4
Hormonal disorders	751	69.0
Genetic predisposition	720	66.2
**Which of the following health complications do you think may result from obesity?**		
Type 2 diabetes	882	81.1
Hypertension	862	79.2
Heart failure	800	73.5
Polycystic ovary syndrome	188	17.3
Sleep apnoea	500	46.0
Asthma	269	24.7
Osteoarthritis	712	65.4
**What do you think are the methods of obesity treatment and management?**		
Increased physical activity	945	86.9
Healthy diet	943	86.7
Regular sleep	393	36.1
Pharmacotherapy of obesity	380	34.9
Surgical operations (bariatric surgery)	561	51.6
Visit to a psychologist/psychodietitian	469	43.1
Visit to a dietitian	739	67.9

### 3.2 Socioeconomic differences in public awareness of causes of obesity

Women, compared to men, more often indicated (*p* < 0.05) a poorly balanced diet, lack of physical activity, mental disorders or experiencing difficult emotions, lack of time for preparing nutritious meals or physical activity, and hormonal disorders as potential causes of obesity (Table 3). Among the respondents aged 60 and older, lack of physical activity and genetic predisposition were noted as causes of obesity more frequently than in other age groups. However, this age group was the least likely (*p* < 0.001) to recognize the link between obesity and mental disorders or experience difficult emotions, and lack of time for preparing nutritious meals and engaging in physical activity. In contrast, awareness of these associations was highest (*p* < 0.001) among the youngest respondents, aged 18 to 29 (Table 3). Awareness of the connection between genetic predisposition and obesity increased with higher BMI categories. Those classified as underweight showed the least awareness, while individuals with obesity showed the highest level of awareness (*p* = 0.01; Table 3).

**Table 3:** Socioeconomic differences in public awareness of causes of obesity in a representative sample of adults in Poland (*n* = 1,088).

**What do you think may be the causes of obesity? (positive answers)**														
	**Poorly balanced diet**		**Lack of physical activity**		**Mental disorders, experiencing difficult emotions**		**Unfamiliarity with the principles of healthy eating**		**Lack of time for preparing nutritious meals and physical activity**		**Hormonal disorders**		**Genetic predisposition**	
**Variable**	***n*** **(%)**	***p***	***n*** **(%)**	***p***	***n*** **(%)**	***p***	***n*** **(%)**	***p***	***n*** **(%)**	***p***	***n*** **(%)**	***p***	***n*** **(%)**	***p***
**Gender**														
Female	457 (77.7)	0.002	500 (85.0)	0.02	358 (60.9)	<0.001	343 (58.3)	0.09	306 (52.0)	0.01	444 (75.5)	<0.001	404 (68.7)	0.06
Male	347 (69.4)		397 (79.4)		214 (42.8)		266 (53.2)		221 (44.2)		307 (61.4)		316 (63.2)	
**Age group [years]**														
18–29	105 (72.4)	0.06	120 (82.8)	**0.001**	101 (69.7)	**<0.001**	90 (62.1)	0.4	91 (62.8)	**<0.001**	100 (69.0)	0.08	89 (61.4)	**<0.001**
30–39	150 (69.8)		160 (74.4)		116 (54.0)		122 (56.7)		116 (54.0)		141 (65.6)		130 (60.5)	
40–49	147 (69.7)		168 (79.6)		108 (51.2)		108 (51.2)		104 (49.3)		134 (63.5)		124 (58.8)	
50–59	153 (80.5)		164 (86.3)		101 (53.2)		105 (55.3)		90 (47.4)		143 (75.3)		133 (70.0)	
60+	249 (76.1)		285 (87.2)		146 (44.6)		184 (56.3)		126 (38.5)		233 (71.3)		244 (74.6)	
**BMI category**														
Underweight	17 (56.7)	0.2	23 (76.7)	0.1	20 (66.7)	0.4	16 (53.3)	0.8	16 (53.3)	0.9	16 (53.3)	0.2	17 (56.7)	**0.01**
Healthy weight	316 (74.2)		340 (79.8)		219 (51.4)		244 (57.3)		207 (48.6)		289 (67.8)		260 (61.0)	
Overweight	309 (75.0)		353 (85.7)		215 (52.2)		231 (56.1)		199 (48.3)		292 (70.9)		286 (69.4)	
Obesity	162 (73.6)		181 (82.3)		118 (53.6)		118 (53.6)		105 (47.7)		154 (70.0)		157 (71.4)	
**Education level**														
Higher	396 (78.3)	**0.002**	431 (85.2)	**0.03**	287 (56.7)	**0.01**	301 (59.5)	**0.03**	257 (50.8)	0.1	376 (74.3)	**<0.001**	342 (67.6)	0.4
Less than higher	408 (70.1)		466 (80.1)		285 (49.0)		308 (52.9)		270 (46.4)		375 (64.4)		378 (64.9)	
**Marital status**														
Single	217 (75.1)	0.7	229 (79.2)	0.4	154 (53.3)	**0.02**	167 (57.8)	0.4	143 (49.5)	**<0.001**	198 (68.5)	0.9	189 (65.4)	0.3
Married	418 (72.4)		483 (83.7)		288 (49.9)		315 (54.6)		262 (45.4)		403 (69.8)		385 (66.7)	
Informal relationship	136 (75.6)		149 (82.8)		112 (62.2)		107 (59.4)		109 (60.6)		120 (66.7)		113 (62.8)	
Other	33 (78.6)		36 (85.7)		18 (42.9)		20 (47.6)		13 (31.0)		30 (71.4)		33 (78.6)	
**Place of residence**														
Rural area	288 (69.4)	0.07	343 (82.7)	0.4	220 (53.0)	0.3	212 (51.1)	0.07	193 (46.5)	0.5	271 (65.3)	0.3	269 (64.8)	0.9
City below 20,000 residents	108 (75.0)		124 (86.1)		68 (47.2)		79 (54.9)		76 (52.8)		100 (69.4)		100 (69.4)	
City from 20,000 to 99,999 residents	158 (74.5)		173 (81.6)		114 (53.8)		131 (61.8)		96 (45.3)		156 (73.6)		140 (66.0)	
City from 100,000 to 499,999 residents	142 (78.0)		143 (78.6)		90 (49.5)		104 (57.1)		91 (50.0)		127 (69.8)		121 (66.5)	
City ≥ 500,000 residents	108 (80.0)		114 (84.4)		80 (59.3)		83 (61.5)		71 (52.6)		97 (71.9)		90 (66.7)	
**Having children**														
Yes	522 (74.4)	0.6	595 (84.8)	**0.007**	347 (49.4)	**0.005**	382 (54.4)	0.2	317 (45.2)	**0.003**	487 (69.4)	0.7	469 (66.8)	0.6
No	282 (73.1)		302 (78.2)		225 (58.3)		227 (58.8)		210 (54.4)		264 (68.4)		251 (65.0)	
**Number of household members**														
1 (living alone)	137 (80.1)	**0.01**	141 (82.5)	0.7	100 (58.5)	0.2	109 (63.7)	**0.01**	77 (45.0)	0.4	120 (70.2)	0.9	117 (68.4)	0.1
2	318 (76.1)		349 (83.5)		210 (50.2)		244 (58.4)		198 (47.4)		288 (68.9)		289 (69.1)	
3 or more	349 (69.9)		407 (81.6)		262 (52.5)		256 (51.3)		252 (50.5)		343 (68.7)		314 (62.9)	
**Occupational status**														
Active	485 (72.6)	0.2	543 (81.3)	0.2	364 (54.5)	0.1	370 (55.4)	0.6	352 (52.7)	**<0.001**	453 (67.8)	0.3	426 (63.8)	**0.04**
Passive	319 (76.0)		354 (84.3)		208 (49.5)		239 (56.9)		175 (41.7)		298 (71.0)		294 (70.0)	
**Self-reported household economic status**														
Good	423 (78.3)	**0.004**	462 (85.6)	0.02	296 (54.8)	0.3	319 (59.1)	0.1	288 (53.3)	**0.005**	391 (72.4)	**0.04**	372 (68.9)	0.1
Moderate	277 (69.4)		321 (80.5)		200 (50.1)		211 (52.9)		172 (43.1)		265 (66.4)		258 (64.7)	
Bad	104 (69.8)		114 (76.5)		76 (51.0)		79 (53.0)		67 (45.0)		95 (63.8)		90 (60.4)	

Statistically significant values are bolded.

Respondents with higher education were more likely (*p* < 0.05) than those with less than higher education to recognize a poorly balanced diet, lack of physical activity, mental disorders or experiencing difficult emotions, hormonal disorders, and unfamiliarity with healthy eating principles as factors contributing to obesity (Table 3). Individuals in informal relationships were more likely (*p* < 0.05) than married and single individuals to attribute obesity to mental disorders or experiencing difficult emotions, and a lack of time for preparing nutritious meals or physical activity as the causes of obesity (Table 3). The same pattern was observed in respondents without children, who were more likely (*p* < 0.05) to recognize these connections than respondents with children. However, the respondents with children identified a lack of physical activity as a contributing factor to obesity more often (*p* = 0.007; Table 3). As household size increased, the ability to identify a poorly balanced diet and unfamiliarity with healthy eating principles as causes of obesity decreased (*p* = 0.01). Specifically, individuals living alone showed the highest awareness of these dietary factors, while those in households of three members or more recognized them the least (Table 3). Occupationally active respondents were less aware (*p* = 0.04) that genetic predisposition could play a role in the development of obesity than occupationally passive respondents. However, they were more likely (*p* < 0.001) to associate a lack of time for preparing nutritious meals or physical activity with obesity (Table 3). Respondents with good self-reported household economic status more often (*p* < 0.05) than those with moderate and bad status indicated a poorly balanced diet, lack of physical activity, lack of time for preparing nutritious meals or physical activity, as well as hormonal disorders as possible causes of obesity (Table 3). There were no significant differences in awareness of causes of obesity by place of residence (Table 3).

### 3.3 Socioeconomic differences in public awareness of complications of obesity

Women were more likely than men (*p* < 0.05) to accurately identify nearly all health consequences of obesity, including type 2 diabetes, heart failure, asthma, sleep apnea, and PCOS (Table 4). Among respondents aged 18 to 29, PCOS was recognized more frequently (*p* < 0.001) compared to other age groups. In contrast, respondents aged 50 to 59 were more likely (*p* < 0.05) to identify sleep apnea and osteoarthritis than those in different age groups (Table 4). The only significant difference (*p* < 0.001) in responses by BMI category was for PCOS, with underweight and healthy weight individuals demonstrating greater awareness than the individuals with overweight and obesity (Table 4).

**Table 4:** Socioeconomic differences in public awareness of health complications associated with obesity in a representative sample of adults in Poland (*n* = 1,088).

**Which of the following health complications do you think may result from obesity? (positive answers)**														
	**Type 2 diabetes**		**Hypertension**		**Heart failure**		**Polycystic ovary syndrome**		**Sleep apnoea**		**Asthma**		**Osteoarthritis**	
**Variable**	***n*** **(%)**	***p***	***n*** **(%)**	***p***	***n*** **(%)**	***p***	***n*** **(%)**	***p***	***n*** **(%)**	***p***	***n*** **(%)**	***p***	***n*** **(%)**	***p***
**Gender**														
Female	511 (86.9)	**<0.001**	472 (80.3)	0.4	448 (76.2)	**0.03**	127 (21.6)	**<0.001**	295 (50.2)	**0.002**	162 (27.6)	**0.02**	395 (67.2)	0.2
Male	371 (74.2)		390 (78.0)		352 (70.4)		61 (12.2)		205 (41.0)		107 (21.4)		317 (63.4)	
**Age group [years]**														
18–29	122 (84.1)	0.4	117 (80.7)	0.3	104 (71.7)	0.08	46 (31.7)	**<0.001**	62 (42.8)	**0.007**	48 (33.1)	0.1	86 (59.3)	**0.008**
30–39	167 (77.7)		164 (76.3)		145 (67.4)		47 (21.9)		109 (50.7)		55 (25.6)		129 (60.0)	
40–49	167 (79.1)		159 (75.4)		152 (72.0)		35 (16.6)		102 (48.3)		49 (23.2)		135 (64.0)	
50–59	160 (84.2)		153 (80.5)		149 (78.4)		28 (14.7)		101 (53.2)		46 (24.2)		143 (75.3)	
60+	266 (81.3)		269 (82.3)		250 (76.5)		32 (9.8)		126 (38.5)		71 (21.7)		219 (67.0)	
**BMI category**														
Underweight	21 (70.0)	0.2	22 (73.3)	0.1	20 (66.7)	0.7	9 (30.0)	**<0.001**	13 (43.3)	0.1	8 (26.7)	0.1	18 (60.0)	0.07
Healthy weight	355 (83.3)		323 (75.8)		314 (73.7)		93 (21.8)		199 (46.7)		120 (28.2)		267 (62.7)	
Overweight	330 (80.1)		336 (81.6)		300 (72.8)		52 (12.6)		174 (42.2)		87 (21.1)		267 (64.8)	
Obesity	176 (80.0)		181 (82.3)		166 (75.5)		34 (15.5)		114 (51.8)		54 (24.5)		160 (72.7)	
**Education level**														
Higher	425 (84.0)	**0.02**	427 (84.4)	**<0.001**	400 (79.1)	**<0.001**	98 (19.4)	0.09	259 (51.2)	**0.001**	146 (28.9)	**0.003**	358 (70.8)	**<0.001**
Less than higher	457 (78.5)		435 (74.7)		400 (68.7)		90 (15.5)		241 (41.4)		123 (21.1)		354 (60.8)	
**Marital status**														
Single	236 (81.7)	0.7	232 (80.3)	0.8	210 (72.7)	0.7	48 (16.6)	0.1	133 (46.0)	0.9	60 (20.8)	0.3	189 (65.4)	0.5
Married	461 (79.9)		454 (78.7)		431 (74.7)		98 (17.0)		260 (45.1)		151 (26.2)		383 (66.4)	
Informal relationship	150 (83.3)		141 (78.3)		127 (70.6)		39 (21.7)		87 (48.3)		47 (26.1)		117 (65.0)	
Other	35 (83.3)		35 (83.3)		32 (76.2)		3 (7.1)		20 (47.6)		11 (26.2)		23 (54.8)	
**Place of residence**														
Rural area	334 (80.5)	0.4	321 (77.3)	0.4	303 (73.0)	0.8	74 (17.8)	0.5	178 (42.9)	**0.04**	103 (24.8)	0.3	269 (64.8)	0.5
City below 20,000 residents	114 (79.2)		117 (81.3)		107 (74.3)		24 (16.7)		62 (43.1)		33 (22.9)		88 (61.1)	
City from 20,000 to 99,999 residents	169 (79.7)		165 (77.8)		157 (74.1)		30 (14.2)		116 (54.7)		45 (21.2)		137 (64.6)	
City from 100,000 to 499,999 residents	147 (80.8)		145 (79.7)		129 (70.9)		31 (17.0)		78 (42.9)		46 (25.3)		123 (67.6)	
City ≥ 500,000 residents	118 (87.4)		114 (84.4)		104 (77.0)		29 (21.5)		66 (48.9)		42 (31.1)		95 (70.4)	
**Having children**														
Yes	569 (81.1)	0.9	549 (78.2)	0.3	524 (74.6)	0.3	109 (15.5)	**0.04**	312 (44.4)	0.2	168 (23.9)	0.4	464 (66.1)	0.5
No	313 (81.1)		313 (81.1)		276 (71.5)		79 (20.5)		188 (48.7)		101 (26.2)		248 (64.2)	
**Number of household members**														
1 (living alone)	141 (82.5)	0.9	137 (80.1)	0.7	128 (74.9)	0.6	21 (12.3)	**0.04**	83 (48.5)	0.8	40 (23.4)	0.8	117 (68.4)	**0.02**
2	337 (80.6)		335 (80.1)		312 (74.6)		66 (15.8)		190 (45.5)		102 (24.4)		290 (69.4)	
3 or more	404 (81.0)		390 (78.2)		360 (72.1)		101 (20.2)		227 (45.5)		127 (25.5)		305 (61.1)	
**Occupational status**														
Active	541 (81.0)	0.9	521 (78.0)	0.2	484 (72.5)	0.3	135 (20.2)	**0.001**	330 (49.4)	**0.004**	173 (25.9)	0.3	434 (65.0)	0.7
Passive	341 (81.2)		341 (81.2)		316 (75.2)		53 (12.6)		170 (40.5)		96 (22.9)		278 (66.2)	
**Self-reported household economic status**														
Good	455 (84.3)	**0.03**	452 (83.7)	**<0.001**	428 (79.3)	**<0.001**	115 (21.3)	**0.002**	272 (50.4)	**0.01**	154 (28.5)	**0.006**	369 (68.3)	0.1
Moderate	313 (78.4)		305 (76.4)		271 (67.9)		55 (13.8)		163 (40.9)		90 (22.6)		251 (62.9)	
Bad	114 (76.5)		105 (70.5)		101 (67.8)		18 (12.1)		65 (43.6)		25 (16.8)		92 (61.7)	

Statistically significant values are bolded.

Respondents with higher education showed significantly more awareness (*p* < 0.05) of type 2 diabetes, hypertension, heart failure, asthma, sleep apnea, and osteoarthritis as possible complications of obesity than those with less than higher education (Table 4). Additionally, there were notable differences in the correct identification of sleep apnea based on place of residence (*p* = 0.04) and in the recognition of PCOS among individuals with and without children (*p* = 0.04), with childless individuals showing greater awareness (Table 4). In terms of household size, respondents living with one other person were the most aware (*p* = 0.02) of the possibility that obesity can cause osteoarthritis. In contrast, respondents in households of three or more members were more likely (*p* = 0.04) to identify a link between PCOS and obesity compared to individuals in smaller households (Table 4). Respondents with an active occupational status were more aware (*p* < 0.05) than those with a passive status that obesity could result in PCOS and sleep apnea (Table 4). Significant differences (*p* < 0.05) were observed in all reported consequences of obesity, except for osteoarthritis, when considering self-reported economic status. Respondents with good financial status demonstrated the most awareness (Table 4). There were no significant differences in the understanding of possible complications of obesity by marital status (Table 4).

### 3.4 Socioeconomic differences in public awareness of obesity treatment and management

Consistent with findings regarding the causes and consequences of obesity, women also outperformed men (*p* < 0.05) in correctly identifying the methods available for the treatment and management of obesity, such as increased physical activity, regular sleep, pharmacotherapy, surgical operations, visits to a psychologist or psycho-dietitian, and visits to a dietitian (Table 5). There were also significant differences (*p* < 0.05) in awareness of those methods across age groups. Specifically, respondents aged 18 to 29 were the most likely to select regular sleep, and a visit to a psychologist or psycho-dietitian as possible treatment and management options. Respondents aged 30 to 39 were the most aware of the possibility of utilizing pharmacotherapy in the treatment of obesity, while those aged 50 to 59 indicated increased physical activity and surgical operations more often than those in other age groups (Table 5). Underweight individuals showed the highest level of awareness (*p* < 0.05) regarding the utility of visits to a psychologist or psycho-dietitian for obesity management. Conversely, individuals with obesity had the least awareness of this option. However, obese individuals, along with individuals with a healthy weight, were more likely to recognize a visit to a dietitian as a management method compared to underweight and overweight respondents (Table 5).

**Table 5:** Socioeconomic differences in public awareness of obesity treatment and management methods among a representative sample of adults in Poland (*n* = 1,088).

**What do you think are the methods of obesity treatment and management? (positive answers)**														
	**Increased physical activity**		**Healthy diet**		**Regular sleep**		**Pharmacotherapy of obesity**		**Surgical operations (bariatric surgery)**		**visit to a psychologist or psychodietitian**		**Visit to a dietitian**	
**Variable**	***n*** **(%)**	***p***	***n*** **(%)**	***p***	***n*** **(%)**	***p***	***n*** **(%)**	***p***	***n*** **(%)**	***p***	***n*** **(%)**	***p***	***n*** **(%)**	***p***
**Gender**														
Female	524 (89.1)	**0.02**	520 (88.4)	0.06	241 (41.0)	**<0.001**	227 (38.6)	**0.006**	334 (56.8)	**<0.001**	302 (51.4)	**<0.001**	428 (72.8)	**<0.001**
Male	421 (84.2)		423 (84.6)		152 (30.4)		153 (30.6)		227 (45.4)		167 (33.4)		311 (62.2)	
**Age group [years]**														
18–29	127 (87.6)	**0.008**	126 (86.9)	0.4	64 (44.8)	**0.007**	59 (40.7)	**<0.001**	70 (48.3)	**0.04**	85 (58.6)	**<0.001**	113 (77.9)	**0.05**
30–39	175 (81.4)		181 (84.2)		85 (39.5)		93 (43.3)		101 (47.0)		102 (47.4)		138 (64.2)	
40–49	176 (83.4)		181 (85.8)		83 (39.3)		73 (34.6)		113 (53.6)		83 (39.3)		143 (67.8)	
50–59	174 (91.6)		172 (90.5)		65 (34.2)		71 (37.4)		116 (61.1)		81 (42.6)		132 (69.5)	
60+	293 (89.6)		283 (86.5)		95 (29.1)		84 (25.7)		161 (49.2)		118 (36.1)		213 (65.1)	
**BMI category**														
Underweight	24 (80.0)	0.5	25 (83.3)	0.3	13 (43.3)	0.7	8 (26.7)	0.4	13 (43.3)	0.6	21 (70.0)	**0.007**	19 (63.3)	**0.04**
Healthy weight	365 (85.7)		365 (85.7)		157 (36.9)		142 (33.3)		215 (50.5)		193 (45.3)		304 (71.4)	
Overweight	362 (87.9)		367 (89.1)		142 (34.5)		144 (35.0)		221 (53.6)		170 (41.3)		259 (62.9)	
Obesity	194 (88.2)		186 (84.5)		81 (36.8)		86 (39.1)		112 (50.9)		85 (38.6)		157 (71.4)	
**Education level**														
Higher	459 (90.7)	**<0.001**	446 (88.1)	0.2	210 (41.5)	**<0.001**	186 (36.8)	0.2	284 (56.1)	**0.005**	248 (49.0)	**<0.001**	352 (69.6)	0.3
Less than higher	486 (83.5)		497 (85.4)		183 (31.4)		194 (33.3)		277 (47.6)		221 (38.0)		387 (66.5)	
**Marital status**														
Single	257 (88.9)	0.6	243 (84.1)	0.4	102 (35.3)	0.9	120 (41.5)	**0.04**	154 (53.3)	0.6	134 (46.4)	**0.02**	191 (66.1)	0.8
Married	499 (86.5)		506 (87.7)		206 (35.7)		182 (31.5)		289 (50.1)		227 (39.3)		394 (68.3)	
Informal relationship	153 (85.0)		159 (88.3)		70 (38.9)		64 (35.6)		98 (54.4)		92 (51.1)		126 (70.0)	
Other	36 (85.7)		35 (83.3)		15 (35.7)		14 (33.3)		20 (47.6)		16 (38.1)		28 (66.7)	
**Place of residence**														
Rural area	360 (86.7)	0.7	344 (82.9)	0.06	140 (33.7)	0.1	133 (32.0)	**0.01**	201 (48.4)	0.2	171 (41.2)	**<0.001**	279 (67.2)	0.4
City below 20,000 residents	126 (87.5)		125 (86.8)		53 (36.8)		43 (29.9)		69 (47.9)		57 (39.6)		94 (65.3)	
City from 20,000 to 99,999 residents	185 (87.3)		190 (89.6)		69 (32.5)		71 (33.5)		117 (55.2)		88 (41.5)		155 (73.1)	
City from 100,000 to 499,999 residents	153 (84.1)		164 (90.1)		71 (39.0)		69 (37.9)		95 (52.2)		71 (39.0)		119 (65.4)	
City ≥ 500,000 residents	121 (89.6)		120 (88.9)		60 (44.4)		64 (47.4)		79 (58.5)		82 (60.7)		92 (68.1)	
**Having children**														
Yes	617 (87.9)	0.2	616 (87.7)	0.2	243 (34.6)	0.2	224 (31.9)	**0.005**	357 (50.9)	0.5	282 (40.2)	**0.008**	472 (67.2)	0.5
No	328 (85.0)		327 (84.7)		150 (38.9)		156 (40.4)		204 (52.8)		187 (48.4)		267 (69.2)	
**Number of household members**														
1 (living alone)	151 (88.3)	0.8	148 (86.5)	0.2	59 (34.5)	0.8	69 (40.4)	0.09	89 (52.0)	0.9	78 (45.6)	0.5	119 (69.6)	0.8
2	363 (86.8)		371 (88.8)		148 (35.4)		131 (31.3)		219 (52.4)		171 (40.9)		280 (67.0)	
3 or more	431 (86.4)		424 (85.0)		186 (37.3)		180 (36.1)		253 (50.7)		220 (44.1)		340 (68.1)	
**Occupational status**														
Active	577 (86.4)	0.6	588 (88.0)	0.1	260 (38.9)	0.02	249 (37.3)	0.04	341 (51.0)	0.7	291 (43.6)	0.7	448 (67.1)	0.4
Passive	368 (87.6)		355 (84.5)		133 (31.7)		131 (31.2)		220 (52.4)		178 (42.4)		291 (69.3)	
**Self-reported household economic status**														
Good	483 (89.4)	**0.02**	489 (90.6)	**<0.001**	212 (39.3)	0.08	214 (39.6)	**0.003**	303 (56.1)	**0.01**	260 (48.1)	**0.004**	391 (72.4)	**0.005**
Moderate	332 (83.2)		333 (83.5)		135 (33.8)		115 (28.8)		190 (47.6)		151 (37.8)		258 (64.7)	
Bad	130 (87.2)		121 (81.2)		46 (30.9)		51 (34.2)		68 (45.6)		58 (38.9)		90 (60.4)	

Statistically significant values are bolded.

Respondents with higher education were more likely than those with lower education (*p* < 0.05) to correctly identify increased physical activity, regular sleep, surgical operations, and visits to a psychologist or psychotherapist as valid treatment and management methods (Table 5). Awareness of the use of pharmacotherapy and visits to a psychologist or psycho-dietitian in the treatment of obesity differed significantly (*p* < 0.05) according to marital status, having children, and place of residence. Respondents without children or those living in large cities were more aware of these treatment options. Single individuals showed greater awareness of pharmacotherapy, while respondents in informal relationships were more likely to recognize visits to a psychologist or psycho-dietitian as possible obesity management approaches (Table 5). Occupationally active respondents were more likely (*p* < 0.05) than those with a passive status to indicate pharmacotherapy and regular sleep as possible obesity treatment and management modalities (Table 5). There were significant differences (*p* < 0.05) in awareness of all treatment and management methods, except regular sleep, based on economic status. Respondents who reported their financial status as good consistently demonstrated greater awareness than those who identified their status as moderate or poor (Table 5). There were no significant differences in awareness of treatment and management methods by household size.

### 3.5 Socioeconomic factors associated with public awareness of obesity as a disease

In the multivariable logistic regression model (Table 6), only the female gender (aOR: 1.62; 95%CI: 1.15–2.27; *p* = 0.005) was associated with higher odds of recognizing obesity as a disease. Age 30 to 39 (aOR: 0.59; 95%CI: 0.37–0.96; *p* = 0.04) was associated with lower odds of recognizing obesity as a disease (Table 6).

**Table 6:** Factors associated with public awareness of obesity as a disease in a representative sample of adults in Poland (*n* = 1,114).

**Do you think obesity is a disease? – “rather yes” or “definitely yes”**						
			**Univariable logistic regression**		**Multivariable logistic regression**	
**Variable**	***n*** **(%)**	***p***	**OR (95%CI)**	***p***	**aOR (95%CI)**	***p***
**Gender**						
Female	516 (87.8)	**0.004**	1.64 (1.17–2.29)	**0.004**	1.62 (1.15–2.27)	**0.005**
Male	407 (81.4)		Reference		Reference	
**Age group [years]**						
18–29	127 (87.6)	0.1	0.96 (0.53–1.73)	0.9	0.87 (0.47–1.58)	0.6
30–39	174 (80.9)		0.58 (0.36–0.93)	**0.03**	0.59 (0.37–0.96)	**0.04**
40–49	178 (84.4)		0.73 (0.44–1.20)	0.2	0.77 (0.46–1.27)	0.3
50–59	156 (82.1)		0.62 (0.38–1.02)	0.06	0.63 (0.38–1.05)	0.08
60+	288 (88.1)		Reference		Reference	
**BMI category**						
Underweight	23 (76.7)	0.2	0.44 (0.17–1.13)	0.09		
Healthy weight	364 (85.4)		0.79 (0.48–1.28)	0.3		
Overweight	342 (83.0)		0.66 (0.40–1.06)	0.09		
Obesity	194 (88.2)		Reference			
**Education level**						
Higher	437 (86.4)	0.2	1.25 (0.90–1.75)	0.2		
Less than higher	486 (83.5)		Reference			
**Marital status**						
Single	232 (80.3)	0.04	0.55 (0.21–1.46)	0.2		
Married	493 (85.4)		0.79 (0.30–2.08)	0.6		
Informal relationship	161 (89.4)		1.15 (0.30–2.08)	0.8		
Other	37 (88.1)		Reference			
**Place of residence**						
Rural area	356 (85.8)	0.5	1.37 (0.82–2.29)	0.2		
City below 20,000 residents	117 (81.3)		0.99 (0.54–1.80)	0.9		
City from 20,000 to 99,999 residents	183 (86.3)		1.43 (0.80–2.57)	0.2		
City from 100,000 to 499,999 residents	157 (86.3)		1.43 (0.78–2.62)	0.3		
City ≥ 500,000 residents	110 (81.5)		Reference			
**Having children**						
Yes	604 (86.0)	0.1	1.29 (0.92-1.82)	0.1		
No	319 (82.6)		Reference			
**Number of household members**						
1 (living alone)	147 (86.0)	0.8	1.17 (0.71–1.92)	0.5		
2	357 (85.4)		1.12 (0.78–1.61)	0.5		
3 or more	419 (84.0)		Reference			
**Occupational status**						
Active	565 (84.6)	0.8	0.95 (0.68–1.34)	0.8		
Passive	358 (85.2)		Reference			
**Self-reported household economic status**						
Good	475 (88.0)	**0.02**	1.69 (1.04–2.75)	**0.03**	1.54 (0.94–2.52)	0.09
Moderate	327 (82.0)		1.05 (0.65–1.71)	0.8	0.97 (0.60–1.58)	0.9
Bad	121 (81.2)		Reference		Reference	

Statistically significant values are bolded.

## 4 Discussion

Recognition of overweight and obesity as a chronic disease remains a contested issue across populations, with significant disparities observed based on gender and age. In the Polish adult population surveyed in this study, 84.8% of respondents acknowledged obesity as a disease, aligning with global trends toward medicalizing obesity. This finding mirrors the shift in clinical and public health narratives as endorsed by the World Health Organization, and reflected in recent definitions by Rubino et al. (2), which classify obesity as a multifactorial, chronic disease affecting physiological, psychological, and metabolic systems. However, not all groups within the study demonstrated equal recognition. Women were significantly more likely than men to perceive obesity as a disease (87.8% vs. 81.4%), while individuals aged 30–39 were the least likely to hold this view (aOR: 0.59; *p* = 0.04), suggesting lingering resistance among younger adults to framing obesity within a medical paradigm.

Public awareness of the causes of obesity was relatively high, particularly regarding lifestyle-related factors. The most frequently cited causes included lack of physical activity (82.4%) and a poorly balanced diet (73.9%). Yet, there were substantial gaps in the understanding of more complex or less visible determinants such as mental health challenges (52.6%), hormonal disorders (69.0%), and genetic predisposition (66.2%). These trends are consistent with other European data (16) and global findings (26), which point to a bias toward behavioral explanations at the expense of biological and psychosocial ones. This incomplete picture undermines the holistic conceptualization necessary for effective interventions. Notably, younger individuals (aged 18–29) were more aware of psychosocial determinants, whereas the older adult (60+) emphasized genetic and physical activity factors but under-recognized emotional and time-related contributors. This suggests generational shifts in perception, perhaps influenced by newer public discourse on mental health and chronic stress. Similarly, those with higher education levels demonstrated greater recognition of multifactorial causes, reinforcing findings from Autret and Bekelman, who documented a strong correlation between education and obesity literacy (6).

When it comes to awareness of complications, most respondents accurately identified type 2 diabetes (81.1%), hypertension (79.2%), and heart failure (73.5%) as consequences of obesity. However, awareness was significantly lower for conditions like sleep apnea (46.0%), osteoarthritis (65.4%), asthma (24.7%), and PCOS (17.3%)—a finding that aligns with prior reports highlighting the public's limited understanding of obesity's less “visible” or sex-specific health outcomes (27). In particular, women consistently showed higher awareness than men across nearly all complications, and education level again emerged as a critical determinant: respondents with higher education recognized six out of seven complications more frequently than their counterparts. These disparities echo findings from other studies, which emphasize the role of disease conceptualization in clinical understanding and stigma reduction (22, 28).

Awareness of treatment options for obesity varied widely. While lifestyle changes such as physical activity (86.9%) and dietary improvements (86.7%) were commonly identified, only 51.6% acknowledged bariatric surgery, and just 34.9% recognized pharmacotherapy as viable treatments. This is particularly concerning in light of the rapid advancements and increased availability of anti-obesity medications, as highlighted by Müller et al. (8) and Berning et al. (29). Despite the evolving pharmacological landscape, a lack of public education persists, potentially hindered by negative social narratives surrounding medicalized treatment, frequently dismissed as a “fashion” or shortcut (30). Again, women, those with higher education, and individuals from households with better self-reported economic status demonstrated higher levels of awareness across all treatment modalities. Urban respondents and those without children were also more informed about psychological and pharmacological interventions, underscoring access and exposure as likely factors. Low awareness of obesity as a disease and its treatment methods may lead to inadequate obesity management.

The social and economic gradient in obesity awareness was clearly evidenced in the study's findings. Respondents with a good economic status were consistently more knowledgeable about the causes, complications, and treatment methods. Conversely, those with moderate or poor financial conditions scored lower across nearly all indicators. This aligns with broader global evidence indicating that socioeconomic status significantly influences both risk exposure and health literacy (16, 27). Particularly striking is the disconnect in pharmacotherapy awareness between high and low socio-economic groups—a gap that may exacerbate health inequalities as novel treatments become more widespread but remain underutilized in lower-income populations. Furthermore, the place of residence was less influential than expected, suggesting that economic status and education may be more potent drivers of awareness than urban vs. rural divides.

Further studies should focus more deeply on gender stereotypes, educational barriers, and access to healthcare as potential reasons for observed differences in public perception of obesity among adults in Poland. Moreover, studies in high-risk populations are also needed.

Considering these findings, it is crucial to reiterate that the costs of obesity are substantial and have a wide economic impact. In 2019, the impact of overweight and obesity was estimated at 2.19% of GDP globally and projected to grow to 3.29% of GDP by 2060, resulting in 5 million deaths (31). Direct costs of the treatment of obesity (including many comorbidities resulting from the disease in question) rise with increasing BMI (32, 33), reaching 0.7% to 17.8% of the total expenditure on healthcare systems, depending on the country (34). Between 2020 and 2050, the OECD countries were predicted to spend on average 8.4% of their total health budgets on the consequences of obesity (35). Moreover, the indirect costs are much higher and can reach even 80% of societal costs (36).

Therefore, investment in prevention is critical and should involve strong education as a primary intervention. Actions should be taken starting from the youngest age, since the costs of overweight and obesity among children are also rising (37).

### 4.1 Practical implications

The findings underscore the pressing need for multidimensional educational interventions aimed at enhancing public literacy around obesity, particularly its recognition as a chronic disease and awareness of its less visible complications and evidence-based treatment options. Given the persistent knowledge gaps among certain demographic groups—especially men, individuals with lower educational attainment, and those in poorer economic conditions—targeted campaigns are essential to promote informed decision-making and reduce stigma. Policymakers should prioritize integrating obesity education into national health strategies, with a specific focus on the younger adult population and the socioeconomically disadvantaged, ensuring access to accurate information on modern therapeutic modalities such as pharmacotherapy and psycho-dietetic support. This approach can strengthen population-level prevention efforts and improve the uptake of effective, individualized obesity treatments. New subject “health education” that will be introduced in school in Poland starting from September 2025 should pay particular attention to building awareness on overweight and obesity among school-aged children and adolescents.

### 4.2 Limitations

This study has several limitations. Firstly, it included only adults who were pre-registered with the research panel and had Internet access, which could restrict the generalizability of the findings to the general population. Additionally, the CAWI methodology is prone to response bias. All data, including anthropometrics, education, and economic status, were self-reported by the study participants and not independently verified by the authors. However, self-reported anthropometrics data are strongly correlated with actual measurements. Furthermore, the study questionnaire was specifically developed by the authors for this study and was therefore not formally validated. Lastly, the study focused solely on the seven most common causes, complications, and treatment and management strategies of obesity, limiting its scope.

## 5 Conclusions

This study reveals that while the majority of Polish adults recognize obesity as a chronic disease, considerable disparities persist in their awareness of its causes, complications, and treatment methods. Lifestyle-related factors, such as physical inactivity and poor diet, are widely understood; however, knowledge about psychosocial and medical contributors remains insufficient. Similarly, the understanding of obesity-related complications and treatment options, especially pharmacological and psychological interventions, is unevenly distributed across demographic and socioeconomic groups. These findings underscore the urgent need for targeted educational campaigns and health policy interventions that aim to enhance obesity literacy, reduce stigma, and improve access to diverse treatment modalities. Future public health strategies should prioritize comprehensive, equitable approaches that consider the nuanced determinants of health knowledge and behaviors within the population.

## Data Availability

The raw data supporting the conclusions of this article will be made available by the authors, without undue reservation.
